# Diagnostics Strategies with Electrochemical Affinity Biosensors Using Carbon Nanomaterials as Electrode Modifiers

**DOI:** 10.3390/diagnostics7010002

**Published:** 2016-12-26

**Authors:** Susana Campuzano, Paloma Yáñez-Sedeño, José M. Pingarrón

**Affiliations:** Departamento de Química Analítica, Facultad de CC. Químicas, Universidad Complutense de Madrid, E-28040 Madrid, Spain; susanacr@quim.ucm.es (S.C.); yseo@quim.ucm.es (P.Y.-S.)

**Keywords:** electrochemical affinity biosensors, diagnosis, carbon nanostructures, graphene, oligonucleotides, proteins, antibodies

## Abstract

Early diagnosis is often the key to successful patient treatment and survival. The identification of various disease signaling biomarkers which reliably reflect normal and disease states in humans in biological fluids explain the burgeoning research field in developing new methodologies able to determine the target biomarkers in complex biological samples with the required sensitivity and selectivity and in a simple and rapid way. The unique advantages offered by electrochemical sensors together with the availability of high affinity and specific bioreceptors and their great capabilities in terms of sensitivity and stability imparted by nanostructuring the electrode surface with different carbon nanomaterials have led to the development of new electrochemical biosensing strategies that have flourished as interesting alternatives to conventional methodologies for clinical diagnostics. This paper briefly reviews the advantages of using carbon nanostructures and their hybrid nanocomposites as electrode modifiers to construct efficient electrochemical sensing platforms for diagnosis. The review provides an updated overview of some selected examples involving attractive amplification and biosensing approaches which have been applied to the determination of relevant genetic and protein diagnostics biomarkers.

## 1. Introduction

Rapid diagnosis of diseases is a critical determinant for the initiation of appropriate treatments. Compared to conventional methods, which should be applied in laboratories by qualified personnel and are time consuming, biosensors are able to diagnose accurately and rapidly at the point of care for patients. Fulfilling these objectives, a variety of biosensors utilizing different manufacturing technologies, detection strategies, or materials for biorecognition have been reported so far for the diagnosis, prognosis and treatment of diseases. Sensitive and selective detection of biomarkers is a safe way to perform this task. In a research area of increasing importance, the observation that many diseases are associated with specific biomarkers, and that the level of biomarkers in biological fluids can vary depending on different disease conditions and stages [1], has opened the door to obtaining relevant results in this field.

In an almost parallel mode, nanotechnology has provided a wealth of diverse nanoscaffolds that can be used to support biomolecules/analytes on electrode surfaces due to their potential features. Biomolecules immobilization onto nanostructured electrochemical transducers reduces diffusion limits and maximizes the surface area, increasing the bioreagents’ loading. It is important to note that direct adsorption onto bulk materials may result in biomolecule denaturation and loss of bioactivity, while nanosized materials not only show a strong tendency to adsorb biomolecules but also retain their bioactivity [2]. Furthermore, nanomaterials can offer various signal amplification routes in electrochemical biosensing as they can be used as electrode materials to construct sensing platforms, carriers for signal elements, tracers based on their direct electrochemistry, separators and collectors, catalysts, mediators to regulate the electron transfer process or a combination of some of these characteristics [3].

## 2. Carbon Nanomaterials as Electrode Modifiers for the Preparation of Electrochemical Biosensors

Carbon nanomaterials (e.g., carbon nanotubes, graphene, fullerenes) are one of the most widely used nanomaterials in electroanalytical applications due to their unique advantages that span several domains, such as high surface-to-volume ratio, highelectrical conductivity, chemical stability, biocompatibility and robust mechanical strength [4]. Carbon has the ability to hybridize into different configurations (sp, sp^2^ and sp^3^) with narrow gaps between their 2s and 2p electron shells. These unique properties enable to prepare high versatile carbon-based nanomaterials which allow the sensitive detection of biological compounds [5]. Sensors involving carbon nanomaterials generally provide higher sensitivities and lower detection limits than their conventional counterparts. Another critical factor that enables functionalization and stable operation of carbon nanostructures in the development of high-performance electrochemical sensors is their morphologies [4]. Most commonly, carbon nanomaterials used to modify electrode surfaces are carbon nanotubes (CNTs) and graphene (GR).

**Figure 1:**
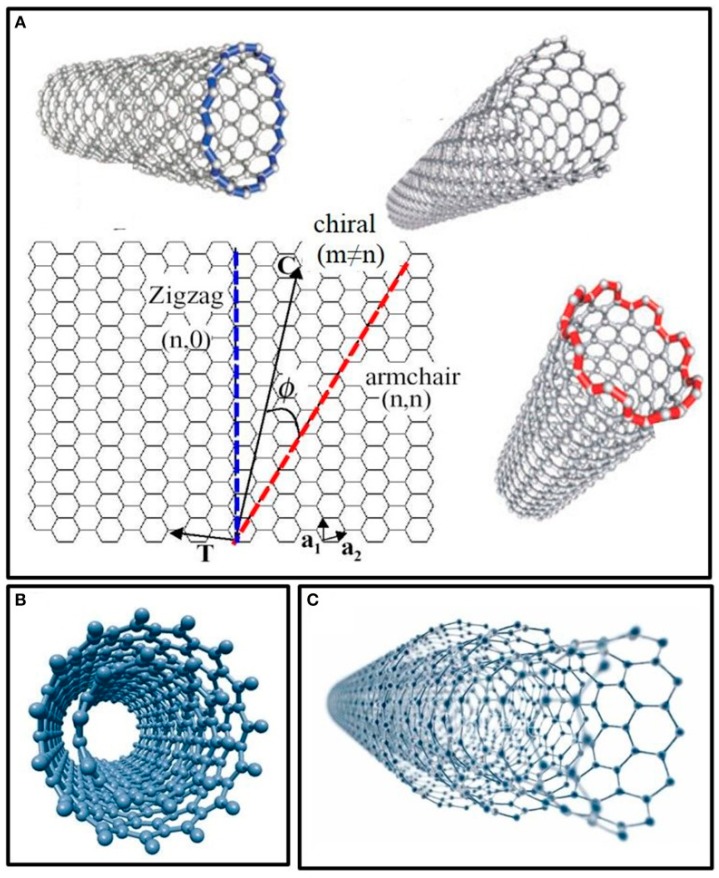
Structure and models of carbon nanotubes in function of their number of walls. (**A**) Single-wall carbon nanotubes (SWCNTs) structures in function of their chirality (zigzag, armchair, and chiral); (**B**) double-walled carbon nanotubes (DWCNTs); and (**C**) multi-walled carbon nanotubes (MWCNTs) made up of several concentric shells. Reprinted from [7] with permission. Figure as originally published in Tîlmaciu, C.-M.; Morris, M.C. Carbon nanotube biosensors. *Front. Chem.* **2015**, *3*, 59. doi:10.3389/fchem.2015.00059.

CNTs have been one of the most promising nanomaterials since their discovery (Iijima, 1991) due to their high electrical conductivity, large specific surface area and excellent chemical and thermal stability [6]. They belong to the fullerene structural family and can be thought of as rolled up graphene sheets. Single-wall carbon nanotubes (SWCNTs) consist of a single graphitic cylindrical layer (φ~0.4–2.5 nm), whereas multi-walled carbon nanotubes (MWCNTs) are constituted by multiply-nested graphene sheets, having variable diameters of up to 100 nm and tunable lengths [4,5,6,7] (See Figure 1). The high electrical conductivity coupled with their small dimensions makes CNTs behave as individual nanoelectrodes. Their efficiency in promoting electron-transfer reactions without using expensive electronic devices is responsible for the signal-to-noise ratio improvement, leading to development of ultrasensitive electrochemical sensors for chemical and biological analytes’ detection [4]. One of the challenges with employing CNTs in biosensors is their inefficient immobilization on the surface to individually disperse nanotubes. Moreover, although carboxylation of CNTs makes them polar and easy to suspend in polar solvents, they have a tendency to form agglomerates due to hydrophobic interactions and van der Waals forces between nanotubes. Different methods have been developed to overcome this issue, such as adsorption on the electrode by drop casting, self-assembled incorporation, covalent grafting to modified electrodes, direct synthesis on the surface and entrapment in conductive organic polymers (COPs) [8]. Another challenge in the development of CNTs-based biosensor devices is their modification by bioreceptors, which can be achieved through chemical attachment of biomolecules to the carboxylic groups of modified CNTs [8]. Recent trends also involve the association of CNTs with other materials with a high number of amine groups on their surface such as dendrimers, which have been successfully applied in the fabrication of sensitive DNA biosensors [5].

On the other hand, graphene, discovered in 2004, is a two-dimensional nanomaterial, a single-atom-thick sheet of hexagonally arrayed sp^2^-bonded carbon atoms which has tremendous potential to modify electrode surfaces due to its unique physicochemical properties [4,9,10,11,12]. The outstanding properties of graphene include large specific surface area, extremely high thermal conductivity, good mechanical strength and high conductivity and electron mobility at room temperature. Graphene facilitates fast electron transfer and provides a non-cytotoxic, large surface for immobilization of biomolecules. Non-covalent interaction such as π–π stacking or hydrogen bonding between graphene/functional graphene and biomolecules makes graphene a promising material for assembling various biomolecules for electrocatalytical sensing applications. However, the present graphene possesses the defect of forming irreversible agglomerates through van der Waals interaction and π stacking, which greatly restricts its further application in the preparation of electrochemical sensors. These limitations have been overcome by using graphene oxide (GO) which possesses enhanced solubility and dispersion ability attributed to the abundant oxygen functional groups (epoxy, hydroxyl, peroxy, carbonyl and carboxyl groups) available on the basal planes and edges [11]. Graphene is often used as the building unit for the preparation of nanocomposites as well as for loading various nanomaterials with different morphologies [13,14]. The resulting hybrid nanomaterials offer specific multi-functions due to the synergistic properties originated from the individual components and their interaction [15].

In the following sections, we provide a broad snapshot of the latest applications of several carbon nanomaterials and their hybrid nanocomposites used as electrode surface modifiers in the development of electrochemical bioaffinity sensors for clinical diagnosis, classified according to the genetic or protein level of the diagnostic targeted biomarker with the aim of conducting a critical evaluation of their characteristics and performance.

## 3. Electrochemical DNA Sensors Involving Carbon Nanomaterials as Electrode Modifiers

Rapid, accurate, cost-effective, and secure microbiological diagnostics of pathogenic DNA stills remains a challenge in the 21st century. The development of extremely sensitive, highly selective, simple, robust, and yet inexpensive biosensing platforms appears thus essential for clinical diagnostics. Direct detection of a genomic DNA sample, without pre-analytical and amplification steps, allowed shortening analysis times and minimizing system complexity and other errors issued from contamination occurring in these further steps. Since accurate diagnostics need specific detection of very small amounts of DNA, there is a need to develop simple label-free DNA hybridization platforms able to perform highly sensitive and specific direct analyses [16]. Moreover, DNA sensors are widely used in diagnostic tests for early cancer and mutation detection, analysis of gene sequences, forensic investigation and assessment of medical treatment [17,18].

Electrochemical sensors possess several characteristics which make them extremely attractive for DNA biosensing in comparison with other methodologies. Fast detection times, high sensitivity (down to several fM and in some cases, aM) and specificity (even for single-base mismatched sequences), easy handling, relatively cheap cost, easy integration into portable platforms, low power consumption and multiplexing capabilities are intrinsic advantages of electrochemical DNA biosensors [3,11,17,18]. They combine the specificity of DNA hybridization with the sensitivity of electrochemical transducers to produce an electroanalytical readout for the analytes. DNA manipulation, including hybridization, ligation or conformational change, is the underlying principle of electrochemical DNA biosensors. They usually involve the immobilization of a specific probe DNA, usually termed capture probe, fully complementary to the target DNA (TD) to be detected, onto the electrode surface and the examination of electrochemical response before and after the hybridization reaction occurred with the TD [10,11,19,20]. Since the performance of electrochemical DNA biosensors largely depends upon the amount and stability of immobilized capture probes at the surface of a sensing device, a variety of approaches for the fabrication of DNA biosensors have been reported using electrodes modified with various materials including carbon nanostructures to overcome the limited capture probe immobilization onto bare electrodes [12,18,21]. The frequently used detection strategies in electrochemical DNA sensors include sandwich-type assays using redox indicators or enzyme labels, assays based on DNA conformational changes and measurement of the electron transfer difference between single (ss-) and double stranded (ds-) DNA [22].

In order to meet growing demand for ultrasensitive DNA detection, many strategies have been developed over the past decade involving sensor modification with different functional materials [3]. In this context, and due to their unique properties, a variety of nanomaterials (gold nanoparticles, AuNPs, CNTs, graphene-related nanomaterials, polymeric NPs) and their nanocomposites [23] have been employed to construct sensing platforms. Their catalytic activity, conductivity and biocompatibility [24], large surface area and abundant binding points, are beneficial for increasing the amount of immobilized DNA probes while retaining their biological activity, and further obtain amplified electrochemical detection signals. Among the variety of nanomaterials used, carbon nanomaterials such as CNTs and graphene have demonstrated fascinating features. While CNTs can provide a high surface area to immobilize DNA molecules and significantly improve the electrochemical properties of the sensors, graphene-related nanomaterials allow direct electrochemical oxidation of DNA bases offering simple detection methods [3]. Moreover, single-stranded DNAs (ssDNA) may be directly immobilized on graphene, GO or carboxyl functionalized GO (CFGO) through π electron interaction as well as covalently through amidation [3,11]. Interestingly, it has been shown that ssDNA exhibits stronger interaction with graphene than dsDNA, and graphene-DNA cannot be easily degraded, the structure remaining stable for a long time [18].

Table 1 summarizes the characteristics of some selected examples of electrochemical DNA biosensors for diagnosis involving the use of carbon nanomaterials. They are critically discussed in the following sections with the aim of showing the enormous potential of DNA electrochemical devices in the field of medical and clinical diagnostics. Furthermore, electrochemical detection is fully compatible with cost-effective hand held microfluidic platforms that bring the possibility of manipulating tiny amounts of samples (<µL) for fast analysis [16].

### 3.1. Electrochemical DNA Sensors Using CNTs

**Figure 2:**
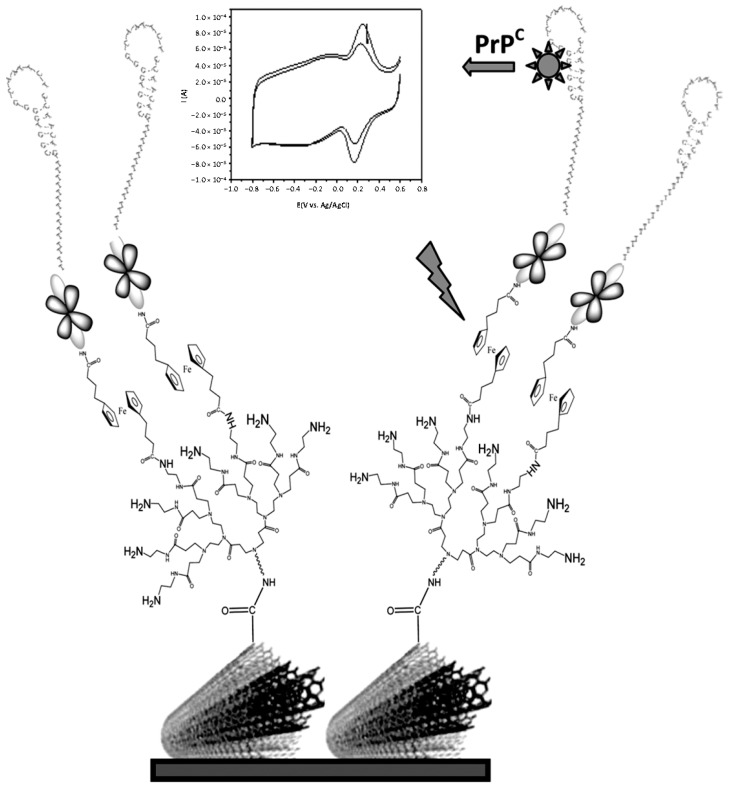
Schematic display of the aptamer biosensor developed for the determination of PrP^C^ using MWCNTs-PAMAM-Fc-biotin-streptavidin bioscaffolds. Reprinted from [26] with permission. PAMAM, polyamidoamine.

Prion proteins are responsible for the transmissible spongiform encephalopathies (TSEs), a group of fatal neurodegenerative diseases including Creutzfeldt-Jakob disease in human and spongiform encephalopathy in animals [25]. These highly contagious diseases are caused by transformation of cellular prions (PrP^C^) into their infectious isoform PrP^Sc^, which are resistant to protease digestion and have a tendency to form large aggregates and form amyloid plaques in brain of mammals. Miodek et al. [26] developed an electrochemical aptasensor for human PrP^C^ protein determination using MWCNTs modified with dendrimers (Figure 2). This approach involved covalent attachment of polyamidoamine (PAMAM) G4 dendrimer to the MWCNTs adsorbed on the surface of a gold electrode. Subsequently, the redox marker ferrocene (Fc) modified with two phtalamido groups (Fc(NHP)_2_) was covalently attached to the PAMAM G4 by the amide link, following by covalent bonding of biotin hydrazide on the remaining phtalymidyl esters of the Fc moieties. Biotinylated aptamers were finally immobilized after streptavidin binding to the biotinylated bioscaffold. Electrochemical signals were obtained by cyclic voltammetry (CV) with the peak currents corresponding to the ferrocenyl redox marker incorporated between the dendrimers and aptamers interlayer. The peak current decreased with the PrPc concentration which was attributed to the lower electron transfer from the ferrocenyl group to the surface due to the decrease of permeability of the sensing layer caused by attachment of the prion proteins to the surface. A LOD of 0.5 pM and a wide detection linear range from 1 pM to 10 μM were achieved for prion protein. The feasibility of this approach for detection of PrP^C^ in spiked blood plasma was also demonstrated.

**Figure 3:**
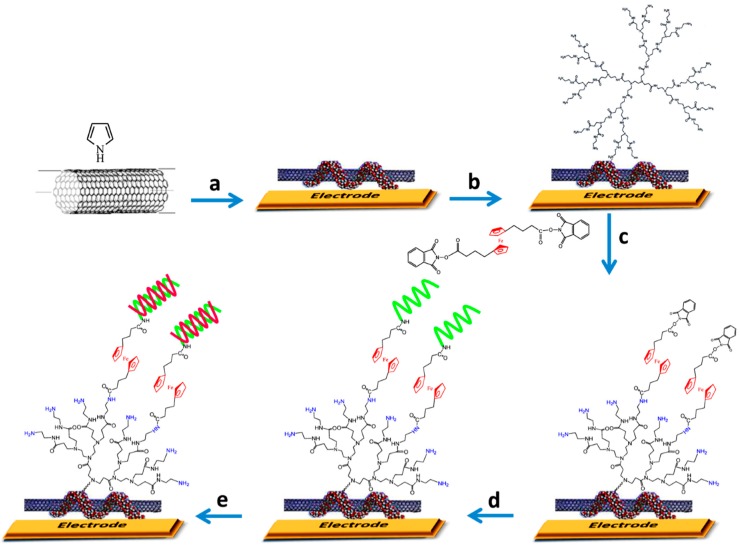
Schematic representation of the steps involved in the biosensor developed for *Mycobacterium tuberculosis rpoB* gene using MWCNTs-PPy-PAMAM-Fc composite nanomaterial. Steps: (**a**) electropolymerization by cycling potential; (**b**) dendrimers attachment through electrochemical oxidation of PAMAM’s G4 amines; (**c**) Covalent attachment of ferrocene; (**d**) covalent attachment of DNA (wavy lines in green), bearing amine in 5′-position, in PBS at room temperature; (**e**) hybridization reaction with the DNA target (wavy lines in red). Reprinted from [8] with permission.

Same authors developed an electrochemical DNA sensor for *rpoB* gene of *Mycobacterium tuberculosis* using composite nanomaterials formed with MWCNTs coated with polypyrrole (PPy) and redox PAMAM dendrimers on gold electrodes [8]. The nanocomposite MWCNTs-PPy was formed by wrapping the PPy film on MWCNTs during electrochemical polymerization of pyrrole on the gold electrode. The MWCNTs-PPy layer was modified with PAMAM G4 with covalent bonding by electro-oxidation method, and Fc functionalized with two activated carboxylic groups was then attached to the surface as a redox marker. A specific amino terminated DNA capture probe was further immobilized on the remaining phtalymidyl esters of the Fc moieties (Figure 3). The diminution in the SWV signal of Fc was used to follow the hybridization process. This biosensor provided linear ranges from 1 fM to 10 pM and from 1 fM to 100 fM for 15 and 75 nucleotides synthetic targets, respectively, a LOD of 0.3 fM for both target DNAs, ability for sensing the target gene in real PCR samples, and suitability for detecting sequences with a single nucleotide polymorphism (SNP) responsible for resistance to rifampicin drug. This performance showed the potential application of this methodology in pathogen diagnostics and therapeutics.

**Figure 4:**
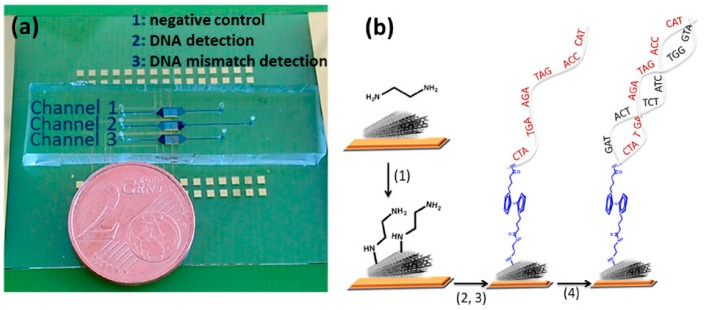
(**a**) Schematic display of the microfluidic multiplexed electrochemical platform developed for specific genetic analysis of *Mycobacterium tuberculosis* and Hepatitis C DNA; (**b**) schematic illustration of the chemical patterning (steps 1, 2, and 3) used for the preparation of the biosensor based on modified ferrocene, as redox marker. Reprinted from [16] with permission.

Zribi et al. [16] developed a microfluidic-multiplexed electrochemical platform for direct impedimetric DNA detection without any amplification of Hepatitis C viral DNA and *Mycobacterium tuberculosis* genomic DNA in clinical isolates. The developed monolithic chip integrated three independent fluidic polydimethylsiloxanes (PDMSs) channels, each containing one electrochemical chamber with three gold electrodes. The working electrode was functionalized with MWCNTs and Fc (Figure 4). Through electrochemical impedance spectroscopy (EIS) measurements in the presence of [Fe(CN)_6_]^4−/3−^ probe, the resulting miniaturized microfluidic device allowed a LOD of 7 fM for a synthetic ss-oligonucleotide from Hepatitis C virus with a large dynamic range from 0.1 fM to 1 pMas as well as a selective direct detection of a *Mycobacterium tuberculosis* (H37Rv) rpoB allele in DNA extracted from clinical isolates without PCR amplification.

### 3.2. Electrochemical DNA Sensors Using Graphene

*Breast cancer 1* (*BRCA1*) gene is a human caretaker gene that is expressed in breast cells and other tissues. Hundreds of mutations in the *BRCA1* gene have been identified and associated with an increased risk of cancer. A graphene-based electrochemical DNA sensor for breast cancer-related *BRCA1* gene detection was developed by Rasheed et al. [18]. The DNA sensor used a sandwich detection strategy, in which specific capture and reporter probes hybridized to target probe DNA on a graphene oxide-modified glassy carbon electrode (GO-GCE). Amino terminated capture and reporter probes were covalently immobilized using 1-*Ethyl*-3-(3-dimethylaminopropyl)carbodiimide/N-Hydroxysuccinimide (EDC/NHS) chemistry on GO-CGE and *O*-(3-carboxypropyl)-*O*′-[2-(3-mercaptopropionyl-amino)ethyl]-polyethylene glycol (CPEG)-modified AuNPs, respectively. The oxidation of conjugated AuNPs was measured by chronoamperometry at +1.1 V vs. SCE and used for determination of the target DNA. This approach provided a linear response with respect to the logarithmic concentration of the target DNA in the 1 fM to 1 nM range and a LOD of only 1 fM (5.896 fg·mL^−1^).

On the other hand, 93%–100% of worldwide invasive carcinomas have been shown to be associated with a limited spectrum of human papillomavirus (HPV) types (a group of non-enveloped, double stranded DNA virus), mostly HPV16 and 18 [10,27,28]. Huang et al. [10] developed an ultrasensitive electrochemical DNA biosensor for HPV detection by immobilizing a specific capture probe (CP) on a GCE modified with GR/Au nanorod/polythionine (GR/AuNR/PT). A sandwich hybridization format between the target DNA (TD, a 34-base fragment specific from *HPV-16* gene long terminal repeat sequences), the capture probe on the electrode surface, and two auxiliary probes (AP1 and AP2) used to long-range self-assemble DNA nanostructure, was used. The differential pulse voltammetry (DPV) signal of [Ru(phen)_3_]^2+^ selected as an electrochemical indicator intercalated in the long dsDNA structure was employed to monitor the hybridization event and determine the target DNA concentration. This label-free DNA biosensor displayed excellent performance for HPV DNA detection over the range from 1.0 × 10^−13^ to 1.0 × 10^−10^ M with a LOD of 4.03 × 10^−14^ M and demonstrated applicability to the analysis of serum samples.

*Staphylococcus aureus* (*S. aureus*) is considered an important factor of infection either acquired from community or hospital infections. It may also lead to serious complications, such as pneumonia, septicemia, arthritis and osteomyelitis [29]. A rapid diagnosis of *S. aureus* infection was reported by amplification of *nuc* gene which encodes the thermonuclease enzyme [30]. A zirconia (ZrO_2_)/graphene nanocomposite was electrodeposited on the surface of carbon ionic liquid electrode (CILE) to construct an electrochemical DNA biosensor for *S. aureus nuc* gene determination [15]. Specific phosphorylated single-stranded DNA (ssDNA) probe sequences at the 5′-end were immobilized on the surface of ZrO_2_/GR/CILE due to the strong affinity between ZrO_2_ and phosphate groups. Hybridization with the target was followed by the decrease of the electrochemical response of methylene blue (MB) used as electrochemical indicator due to the hindered interaction of MB with the guanine residues of the probe ssDNA sequence. Under the optimal conditions, the reduction peak current of MB (measured by DPV) was inversely proportional to the concentration of *S. aureus nuc* gene sequence in the range from 1.0 × 10^−13^ to 1.0 × 10^−6^ mol·L^−1^ allowing detection of 3.23 × 10^−14^ mol·L^−1^ (3σ). The electrochemical DNA sensor demonstrated good selectivity to various mismatched ssDNA sequences and applicability to amplification products of *S. aureus nuc* gene sequence obtained by PCR.

*Vibrio parahaemolyticus* is a gram-negative bacterium distributed throughout the estuarine environment considered responsible of acute gastroenteritis and septicemia in humans [31]. The infection with this bacterium, which usually occurs when consuming raw or undercooked seafood, can be diagnosed using as target the *thermolabile hemolysin* (*tlh*) gene since it exists in all the strains identified so far [32]. Yang et al. [11] developed a sensitive electrochemical DNA biosensor for *tlh* gene detection using a SWCNTs-carboxyl functionalized GO-modified GCE (SWCNTs/CFGO/GCE) and specific amino terminated capture DNA probes immobilized covalently using EDC/NHS chemistry onto the electrode surface. The electrochemical responses obtained by DPV in the presence of [Fe(CN)_6_]^3−/4−^ were employed to monitor the hybridization event. With this approach, the target DNA sequence could be detected in a concentration range from 1 × 10^−6^ to 1 × 10^−13^ mol·L^−1^ and with a LOD of 7.27 × 10^−14^ mol·L^−1^ (S/N = 3).

**Figure 5:**
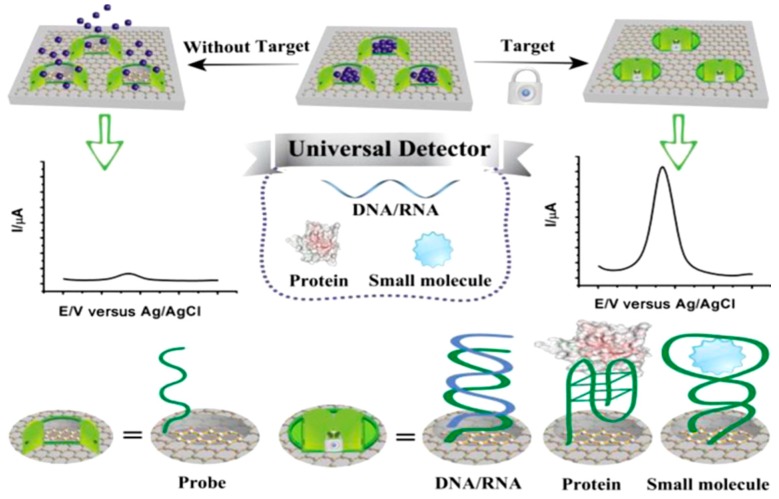
Schematic illustration of the target-responsive encapsulation assay (TRE) electrochemical biosensor for determination of various classes of biologically relevant molecules. Reprinted from [21] with permission.

Wu et al. [21] developed an ultrasensitive universal electrochemical detection system based on a target-responsive encapsulation assay (TRE) using multifunctional hybrids of GR and mesoporous materials (MSGNs) and single-stranded DNA probes as the pore-caps for various classes of biologically relevant molecules. The concept is based on the loading of electroactive molecules (MB and Fc) on MSGNs and the locking of the pores and preventing the signal-reporter molecules from escape by target-induced conformational change of the tailored DNA caps. Only in the presence of the target analytes does the intelligent “gatekeeper” switch its conformation close to the pores and hence leading to an increase in the measured analytical signal (Figure 5). Furthermore, the MSGNs constitute a versatile matrix which can incorporate various redox-active molecules into the mesopores thus making feasible the simultaneous evaluation of different targets (small molecules, nucleic acids and proteins). This label-free scaffold was applied to the determination of DNA sequences correlated to Alzheimer, thrombin, ATP, and Hg^2+^ and Ag^+^ ions.

**Figure 6:**
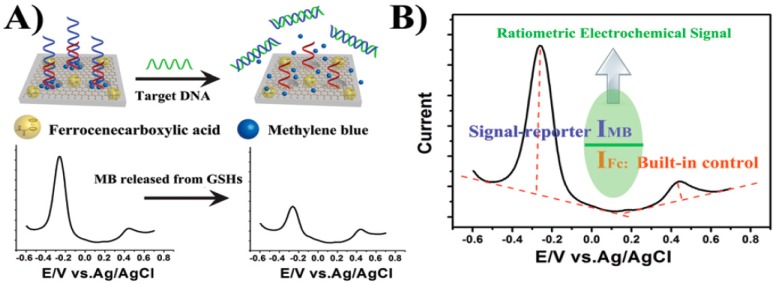
Schematic illustrations of the ratiometric electrochemical DNA sensor developed (**A**); and the electrochemical signal obtained (**B**). Reprinted from [12] with permission.

A novel ratiometric electrochemical nanosensing platform was developed using GR and mesoporous hybrid nanomaterials (termed graphene@mesoporous silica hybrids, GSHs)-modified GCE for detection of the mutated *apolipoprotein E* gene associated with Alzheimer’s disease [12]. The GSHs served as nanoreservoirs for the loading of MB electro-active molecules. Ferrocene carboxylic acid was covalently conjugated to the as-synthesized nanomaterials (Fc–GSHs) and used as the inner reference molecules to provide the built-in control for indicating the amount of GSHs. A duplex DNA probe was connected to the surface of GSHs, preventing the leakage of loaded caged electrochemical reporter (MB) whereby molecules are released only upon target DNA-induced opening of the nanopores and generation of a measurable “on–off” current. Therefore, the electrode material (GSHs) is designed for encapsulating electro-active molecules and accelerating electron transfer by incorporating the various functions of both mesoporous silica and graphene into a single hybrid nanostructure without losing the individual properties of each component. The ratiometric output signal was obtained by calculating the ratio of peak current generated by caged signal-indicator MB and built-in control Fc, both measured by DPV, as it is shown in Figure 6. The self-calibrated characteristic of ratiometric electrochemical method with the ratio of dual-signaling responses as the output signal will rule out the dependence on the concentration of graphene based nanocapsules and substantially overcome intrinsic systematic errors commonly associated with signal-off and nanomaterial-based sensors derived from environmental and personal factors, enabling the detection method to be relatively reliable and reproducible and improving the accuracy of target detection.

## 4. Electrochemical Immunosensors Involving Carbon Nanomaterials as Electrode Modifiers

Electrochemical immunosensors have been extensively used for the determination of diagnostics biomarkers because of the interesting advantages they offer in terms of inherent high sensitivity and selectivity, great precision and accuracy, low cost, minimum sample requirements, simplicity of operation and possible integration into compact analytical devices as well as demonstrated suitability for clinical analysis [33,34,35,36,37,38,39,40]. In the following sections, the most interesting characteristics of selected approaches using carbon nanomaterials as modifiers of electrochemical immunosensing platforms will be briefly discussed.

### 4.1. Electrochemical Immunosensorsusing CNTs

Due to the excellent properties for electron-transfer reactions and the high stability and large surface area per volume provided by CNTs, they have been frequently used for improvement of immunosensing systems upgrading transductor responses in conjunction with electrochemical techniques. The incorporation of specific functional groups to CNTs’ surface enables immobilization of immunoreagents and expands the applications in the medical and clinical fields for diagnosis and treatment of diseases. CNTs immunosensors can provide sensitive and fast responses in the detection of specific biomarkers at low concentrations in complex samples, and the combination with other materials such as AuNPs, conducting polymers, or distinct carbon nanomaterials may improve biocatalytic activities and stability.

Owing to the high number of applications, this section has been limited to immunosensors for cancer and cardiovascular diseases’ diagnosis. For more information, Table 2 was included summarizing the analytical properties of some recent approaches.

Detection of tumor markers plays an important role in screening, diagnosing and evaluating the prognosis of cancer diseases, and herein, electrochemical immunosensors have demonstrated a high degree of reliability and robustness. Chikkaveeraiah et al. [41] reviewed applications of electrochemical immunosensors for detection of cancer protein biomarkers with abundant examples referring to devices using CNTs. An illustrative recent example is the preparation of an impedimetric immunosensor using a MWCNTs-ionic liquid electrode (MW-CILE) with electrodeposited AuNPs for the determination of human epidermal growth factor receptor 2 (HER2). This tyrosine kinase receptor belongs to the epidermal growth factor receptor (EGFR) family involved in cellular signaling pathways frequently associated with different diseases such as breast cancer, where over expression of HER2 is observed [42]. The developed configuration involved the use of additional AuNPs immobilized onto AuNPs/MW-CILE through 1,6-hexanedithiol to improve the extent of immobilization and stability of Herceptin antibody. Monitoring of immunocomplex formation with different amounts of HER2 was made via the changes of impedance responses, these providing linear increases in a clinically useful concentration range [43]. Another representative immunosensor was constructed for carcinoma antigen-125 (CA-125), a member of the mucin family of glycoproteins that constitutes a standard clinical biomarker associated with gynecological malignancies [44]. For its sensitive detection, an electrochemical platform using MWCNTs embedded highly oriented ZnO nanowires was constructed [45]. Interestingly, the bioelectrode was prepared by a one-step calcination process at the optimum temperature to avoid decomposition of MWCNTs but enough to create functional groups on MWCNTs-ZnO surface for covalent immobilization of the antibody. A label-free detection scheme using DPV was designed using [Fe(CN)_6_]^3−/4−^ as a redox probe, this providing good sensitivity. Moreover, the fabricated immunosensor showed good stability, reproducibility and acceptable selectivity.

Prostate specific antigen (PSA) is an androgen-regulated serine protease whose levels have been identified as a reliable tumor marker for the early diagnostics of prostate cancer [46]. A variety of electrochemical immunosensors have been developed for PSA detection characterized by relevant analytical advantages such as high sensitivity and simple instrumentation. Salimi et al. [47] described an electrochemical immunosensor for PSA involving immobilization of anti-PSA onto a MWCNTs/IL composite, with IL = 1-butyl-methylpyrolydinium bis(trifluoro-methylsulfonyl)imide [C4mpyr][NTf2], with adsorbed thionine (THI) as redox system for the electrochemical probe. A sandwich configuration was established using a detection antibody labeled with peroxidase and H_2_O_2_ as the enzyme substrate. This strategy displayed a surface-controlled electrode process with an electron transfer constant of 6.5 s^−1^, providing good sensitivity. The same group reported the preparation of a GCE modified with a MWCNTs/chitosan (Chit)/IL nanocomposite to immobilize AuNPs-PAMAM dendrimer conjugates with covalently attached anti-PSA capture antibody and THI as the redox mediator. As in the previous approach, a sandwich immunoassay using HRP-Ab2 was carried out, and PSA was determined by electrochemical detection of H_2_O_2_. However, a higher sensitivity was found in this latter case due to the increased number of covalently attached anti-PSA molecules and the observed synergistic effects of AuNPs and PAMAM enhancing the electrocatalytic activity of THI/HRP system toward H_2_O_2_ reduction. Moreover, electrochemical impedance spectroscopy was also applied with good results by using the anti-PSA/AuNPs-PAMAM/MWCNTs/IL/Chit/GCE free-label configuration [48]. More recently, a simple immunosensor for PSA was prepared using MWCNTs-modified GCE as an immobilization platform for anti-PSA. A 1,7-diaminoheptane (DAH) monolayer was assembled first onto GCE to further link carboxylated MWCNTs and capture antibodies. Immunosensing involved signal amplification by means of AuNPs modified with the secondary antibody (Ab2) and 6-ferrocenyl hexanethiol (FcH) acting as a signaling molecule. This approach represents a multiple signal amplification strategy in which functionalized CNTs improved the electron transfer on the electrode surface, while AuNPs acted as carriers for capturing large quantities of Ab2 and FcH [49].

The glycolytic enzyme NSE (neuron specific enolase) is used as a biomarker in cases of small-cell lung cancer (SCLC) where increased levels from the normal value, 9 ng·mL^−1^, can be found [50]. GCEs modified with functionalized SWCNTs were used for immobilizing NSE antigen and to design of a competitive immunoassay with anti-NSE antibody. The electrochemical detection involved the use of AuNPs labeled with alkaline phosphatase (AP) conjugated-secondary antibody (AP-anti-IgG/AuNPs). In this way, dual signal amplification was attained: firstly, SWCNTs enhanced the electrochemical responses at the electrode and also presented abundant antigen domains for recognition of anti-NSE and, secondly, AP-anti-IgG/AuNPs exhibited high catalytic activity toward α-naphthyl phosphate used as the enzyme substrate, significantly amplifying the amperometric responses [51]. SWCNTs were also used for the development of a sandwich-type immunosensor for interleukin-6 (IL-6). This multifunctional cytokine, characterized as a regulator of immune and inflammatory responses, is overexpressed in several types of cancer, including head and neck squamous cell carcinoma (HNSCC). Mean serum IL-6 in patients with HNSCC is ≥20 pg·mL^−1^ compared to ≤6 pg·mL^−1^ in healthy individuals. In this strategy, SWCNTs forests with attached capture antibodies (Ab1) for IL-6 were used in an electrochemical immunoassay protocol involving secondary antibodies (Ab2) attached to HRP multienzyme labeled-MWCNTs containing 106 HRP labels per 100 nm. The high loading of HRP combined with the electrode nanostructuration allowed a high sensitivity to be attained for the determination of IL-6 in complex biological samples [52].

An original configuration of electrochemical immunosensors for the detection of α fetoprotein (AFP) was described by Lin et al. [53]. AFP is one of the most important tumor biomarkers for the assistant diagnosis of hepatocellular cancer, and its determination has been the subject of numerous electrochemical immunosensors [54]. Oxidized SWCNTs were covalently linked on the internal pore walls of mesoporous silica (MPS) and used to confine anti-AFP antibodies inside the mesopores. Silanol groups on the external surface of MPS were blocked by trimethylchlorosilane (TMCS) whereas those internal were grafted with amino groups using aminopropyltrietoxysilane (APTS). Then, GCE was coated with both GR and the resulting anti-AFP/SWCNTs/TMCS-MPS to prepare an immunosensor through layer-by-layer (LBL) assembly. AFP immuno-conjugation resulted in the increment of spatial blocking and impedance of the immunosensing interface giving rise to a decrease in the peak current with increasing AFP concentration. The two types of carbon nanomaterials used in this configuration contributed to improved sensitivity: SWCNTs inside the mesopores could promote the electron transport through the pore channel, and the external GR film, with a high conductive capacity, could also improve the electrochemical response.

A label-free immunosensor was also fabricated for carcioembryonic antigen (CEA). This is a glycoprotein most often associated with colorectal cancer but also found at elevated levels in patients with lung, ovarian or breast cancers. The normal concentrations of CEA in healthy adults are in the range of 3–5 ng·mL^−1^ although these levels increase up to 10 ng·mL^−1^ in some benign diseases. Positively charged MWCNTs wrapped with poly(diallyl-dimethyl-ammonium) (PDDA) and negatively charged poly(sodium-p-styrene-sulfonate) (PSS) were LBL assembled on a GCE. Further electrodeposition of gold nanoclusters allowed obtaining the Au/PDDA/MWCNTs/(PSS/PDDA/MWCNTs)_2_/GCE platform with a large specific surface for antibodies’ immobilization. Direct immunoassay with anti-CEA immobilized was performed using cyclic voltammetry for CEA detection by measuring the oxidation peak of [Fe(CN)_6_]^3−/4−^ redox probe [55]. Carboxylated MWCNTs/doped nylon 6 (PA6) composite nanofibers were prepared by electrospinning and used as the nanosized backbone for thionine (THI) electropolymerization. The resulting functional material (MWCNTs-PA6-pTHI) served as supporting scaffold for electron transfer promoting and increasing the surface area to immobilize captured antibodies in a large quantity. This configuration was used for developing an electrochemical immunosensor for the determination of tumor suppressor protein (p53), which is related with various types of cancer [56]. A sandwich immunoassay that involved antigen conjugation with immobilized anti-p53 and interaction with a detection antibody labeled with HRP was developed. In this way, addition of hydrogen peroxide produced an amplified electrocatalytic response by reduction of enzymatically oxidized pTHI [57].

Cardiovascular diseases are a major threat to world health, with the diagnosis and prevention of acute myocardial infarction being of paramount importance. Monitoring of cardiac biomarkers such as cardiac troponins (cTn), myeloperoxidase (MPO), myoglobin (Mb), creatine kinase MB (CK-MB), or N-terminal pro-B type natriuretic peptide (NT-proBNP) provides an effective strategy for diagnosis and prevention where electrochemical immunosensors involving carbon nanomaterials have held a starring role for many years [58]. Some examples of recent configurations using CNTs are reviewed below and their analytical characteristics summarized in Table 2.

**Figure 7:**
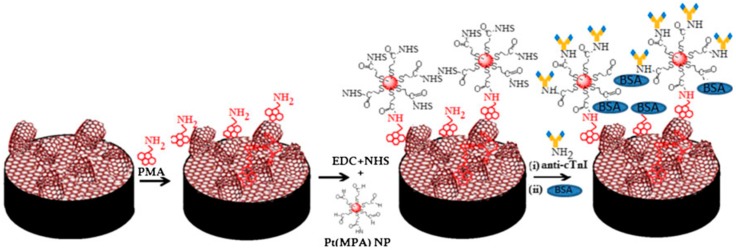
Schematic representation of stepwise fabrication of the anti-cTnI-PtMPA/GR-MWCNTs/GCE immunosensor. Reprinted from [61] with permission.

Cardiac troponins consist of a complex of troponin C (TnC), troponin I (TnI) and troponin T (TnT) that regulates the contraction of striated and cardiac muscle. Both cTnT and cTnI are recommended markers for evaluation of acute coronary syndrome, whereas cTnC is unspecific. The existence of multiple forms of cTnI and their low stability makes the application of analytical procedures difficult. In addition, relatively small differences in cTnI concentrations have been described between health and disease conditions. Levels of cTnI in healthy humans are normally lower than 0.1 ng·mL^−1^ whereas values between 0.1 and 2 ng·mL^−1^ suggest a diagnosis of unstable angina and other heart disorders and levels greater than 2 ng·mL^−1^ indicate an increased risk for future serious heart events [59]. Therefore, monitoring of these biomarkers for diagnostic purposes requires the use of highly sensitive and accurate detection systems. An interesting example is the preparation of an immunosensor for the determination of cTnI by coating a GCE with a GR-MWCNTs hybrid film. This type of composite combines the large specific surface area and three-dimensional framework of CNTs with the high edge-density of GR, and exhibits synergistic effects between both different graphitic nanostructures [60]. In the reported configuration, the resulting hybrid was modified with platinum nanoparticles that were capped with mercaptopropionic acid (MPA) and anchored on the GR-MWCNTs film via the self-assembled cross-linker 1-pyrene-methylamine (PMA) (Figure 7). The resulting surface provided site specific immobilization with high loading of anti-cTnI antibodies for label-free cTnI immunosensing using EIS [61].

A gold electrode modified with polyethyleneimine (PEI) was used to prepare a nanostructured platform with linked CNTs and covalent immobilization of anti-cTnT antibodies. PEI in the branched form provided a high density of amine groups to bind carboxylated CNTs that acted as an electron promoter between the polymer film and solution. In the resulting functionalized surface, anti-cTnT was covalently immobilized to the remaining carboxylate groups of CNTs, and a sandwich type immunoassay involving anti-cTnT-HRP and H_2_O_2_ was employed. Amperometric detection in the absence of redox mediator was used to determine the antigen in a concentration range (0.1–10 ng·mL^−1^) significant for acute myocardial infarction diagnosis [62]. Furthermore, a label-free immunosensor using amine-functionalized CNTs-SPE was also described for the detection of cTnT. The disposable electrode was fabricated using an adhesive carbon ink containing CNTs to form a thin film onto polyethylene terephthalate substrate. Once anti-cTnT antibodies were immobilized, a direct immunoassay was performed with [Fe(CN)_6_]^3−/4−^ redox probe after cTnT incubation [63].

High levels of myeloperoxidase (MPO) in plasma were reported to be a risk factor for early adverse cardiac events such as myocardial infarction [64]. For the sensitive detection of this enzyme, an electrochemical immunosensor was developed involving self-assembly of MWCNTs, THI, AuNPs and Chit multilayers onto GCEs. Anti-MPO was adsorbed on AuNPs, and HRP was employed both as blocking agent for non-specific binding and to amplify the electrochemical responses [65]. A disposable electrochemical MPO immunosensor was also fabricated using indium tin oxide electrode (ITO) modified with a film composed of poly(*o*-phenylenediamine) (*o*PD), MWCNT, the ionic liquid 1-ethyl-3-methylimidazolium bromine ([EMIM]Br, IL), and adsorbed AuNPs. In situ electropolymerization of *o*PD/MWCNTs using the IL as the supporting electrolyte was used to prepare the composite film on the electrode surface. Negatively charged AuNPs were then adsorbed on the modified electrode via amine-gold affinity and used to immobilize MPO antibody. Direct immunoassay was performed to determine MPO with [Fe(CN)_6_]^3−/4−^ label detecting the decreases in current provoked by hindering the electron transfer as the antigen concentration increased [66]. Myoglobin (Mb), cTn I and creatine kinase MB (CK-MB) biomarkers were determined using MWCNTs embedded with SU-8 electro-spun nanofibers. The composite nano-fibers exhibited excellent electrical and transduction properties owing to the ease of functionalization and biocompatibility. The synthesized nanofibers were functionalized with specific antibodies to these protein biomarkers and EIS was used for detection [67].

Increased plasma levels of low density lipoprotein (LDL), especially in the oxidized form (oxLDL), are associated with atherosclerosis. Therefore, circulating oxLDL is commonly recognized as an important predictive marker for risk of cardiovascular events. An immunosensor employing three monoclonal antibodies against oxLDL was proposed to ensure specific antigen binding towards the target. The immunosensor was set-up by self-assembling cysteamine (Cyst) on a gold layer of a disposable SPE. The antibodies were covalently immobilized onto the Cyst/Au surface and the determination of oxLDL was made by EIS and SWV after incubation of the immunosensor into oxLDL solutions for 15 min [68].

An electrochemical immunosensor for myoglobin (Mb) was also developed. Mb regulates the storage and diffusion of oxygen in heart and skeletal muscles. It is a biomarker for muscle injury in patients with chest pain, which is a potential code for heart attack, and it is also considered as the most sensitive marker for myocardial infarction [69]. A label-free impedimetric immunosensor for Mb detection was reported involving immobilization of a monoclonal anti-Mb antibody on a MWCNTs/SPE and direct detection of the antigen by measurement of changes in the charge transfer resistance (R_CT_) with increasing Mb concentration [70]. Netrins are a class of proteins involved in cell migration and axon guidance during development. A class of netrins, netrin1, has shown to be abundantly expressed in atherosclerotic lesions, this giving a new perspective for the prediction of atherosclerosis and other cardiovascular diseases [71]. An immunosensor to determine netrin1 in serum was reported using a GCE modified with MWCNTs, nafionTHI-coated gold nanoparticles (THI/AuNPs), and anti-netrin 1 antibody. The presence of Thi/AuNPs warrants direct and convenient immobilization of the antibody [72].

**Figure 8:**
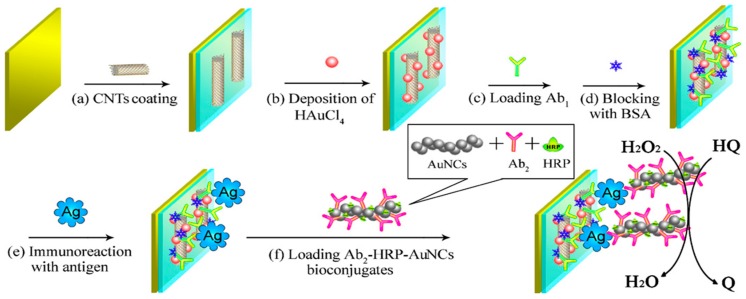
Scheme of the fabrication steps o fNT-proBNP immunosensor using AuNCs-HRP-Ab2 and AuNPs-CNTs nanocomposites. Reprinted from [74] with permission.

Clinical data have also revealed a strong association between the N terminal pro-B-type natriuretic peptide (NT-proBNP) level and the mortality in patients with heart failure [73]. An ultrasensitive electrochemical strategy for NT-proBNP detection was developed using gold nanochains (AuNCs) and HRP complex amplification at a nanostructured gold functionalized CNTs composite. AuNCs enabled the efficient immobilization of biomolecules and acted as conductive centers to facilitate electron transfer [74]. The AuNCs were prepared by using l-ascorbic acid (AA) as a mediator and template. The nanostructured surface enhanced the amount of immobilized primary antibodies (Ab1). More importantly, improved sensitivity could be achieved by introducing the multibioconjugates of AuNCs-HRP-Ab2 onto the electrode surface through sandwich immunoreactions (Figure 8).

### 4.2. Electrochemical Immunosensors Using Graphene

In recent times, GR has stimulated a high amount of electrochemical immunosensors due to its unique properties [75]. In the variety of reported applications, this 2D carbon nanomaterial has proven able to effectively contribute to the development of sensitive detection of biomarkers using different immunoassay strategies because of the high biocompatibility and fast electron transport [76]. Selected applications of graphene-involving immunosensors for detection of cancer and cardiovascular biomarkers are summarized in Table 3.

Among the different types of GR, reduced graphene oxide (rGO) has recently aroused much interest for the fabrication of electrode scaffolds. Its abundant defects and chemical groups facilitate charge transfer and ensure increased electrochemical activity [77]. A representative example is the highly sensitive electrochemical immunosensor proposed by Ma et al. [78] for the detection of nuclear matrix protein 22 (NMP22), a typical bladder cancer biomarker. rGO combined with tetraethylenepentamine (TEPA-rGO) was dropped on the surface of GCE followed by trimetallic nanoparticles conjugated with capture antibody (AuPdPt NPs-anti-NMP22). Covalent functionalization of rGO with TEPA provided rGO stability avoiding agglomeration while keeping its excellent properties. Moreover, the large number of amino groups on the TEPA-rGO/GCE can effectively immobilize AuPdPt NPs-anti-NMP22 conjugates thus enhancing the performance of the immunosensor. Meanwhile, trimetallic NPs could accelerate the electron transfer assisted by the synergistic effect of the different metals, and improve the stability of the electrochemical immunosensor by maintaining the bioactivity of antibodies.

As can be seen in Table 3, label-free electrochemical detection combined with immunosensors using GR has been used frequently for biomarkers determination. A representative design was developed for α-fetoprotein (AFP) by immobilizing anti-AFP onto a nanocomposite consisted of rGO and SnO_2_/AuNPs deposited onto GCE. A simple method for synthesizing SnO_2_/AuNPs was applied based on HAuCl_4_ reduction with SnCl_2_ in the presence of bis(amidoethyl-carbamoylethyl)-octadecylamine (C18N3), a single chain surfactant with multi-amine head groups which acted as an stabilizer of the resulting nanomaterial. In this configuration, peak currents of [Ru(NH_3_)_6_]^3+^ at the immunosensor were measured for the detection of AFP [79]. Electrochemical responses from redox probes included in the electrode platform can also be used for label-free detection. One example is the CEA immunosensor prepared by Gao et al. [80] based on a composite of Nile blue A (NB) and reduced graphene oxide (NB/rGO) with anti-CEA antibody captured by AuNPs. The NB/GO hybrid was prepared by π–π stacking interaction and, then, it was electrochemically reduced in the presence of HAuCl_4_. The resulting anti-CEA-AuNPs/NB/rGO immunosensor took advantage of the good electrochemical behavior of NB present in the electrode. Measurement of the decrease in NB currents due to the immunocomplex formation were used to determine CEA, and the method provided good results in the application to saliva samples. A different approach for determining this same cancer biomarker was reported by Huang et al. [75]. The proposed design involved the use of AuNPs coated on GRto prepare an electrode scaffold for immobilization of the capture anti-CEA antibody (Ab1) and the implementation of a sandwich-type immunoassay with signal amplification by means of an Ag/Au NPs-coated GR tracer to label the secondary antibody (Ab2). 1,5-diaminonaphthalene (DN) was adsorbed onto GR and the amino groups were used to coat AuNPs or Ag/AuNPs and conjugated with Ab1 or Ab2. The electrochemical activity observed by cyclic voltammetry for the Au/AgNPs-DN-GR label, corresponding to direct oxidation of Ag to Ag_2_O and further reduction, was used to follow the immunocomplexation event and to determine CEA in a wide concentration range.

For the sensitive and label-free detection of the prostate specific antigen (PSA), a 3D electrochemical immunosensor involving highly conductive GR-AuNPs composite-modified GCEs was also designed. Three-dimensional nanomaterial showing a shape of a crumpled GR ball decorated with AuNPs was obtained by aerosol spray pyrolysis. Compared with the equivalent immunosensor based on 2D GR-gold nanocomposites, the crumpled 3D exhibited much higher current change rates as corresponded to the larger surface area, higher loading of AuNPs conjugated to antibodies, and superior catalytic ability [76].

**Figure 9:**
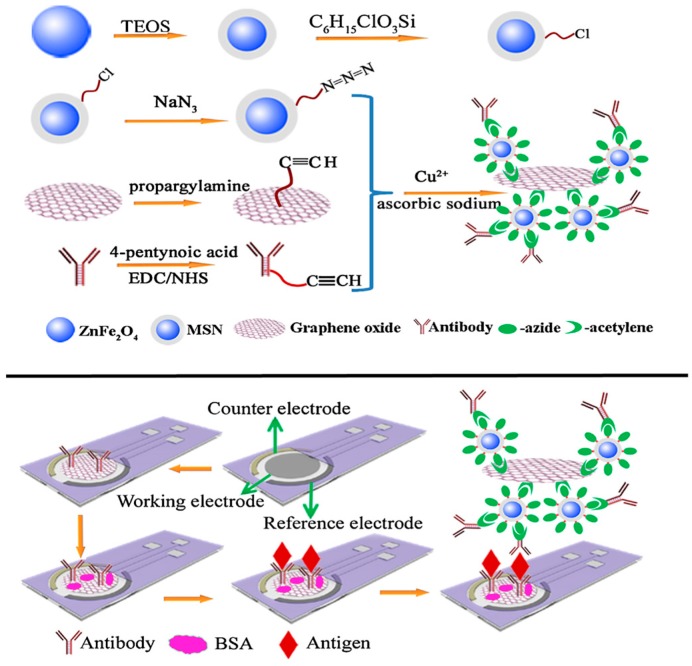
Schematic representation of the CA-15-3 immunosensor preparation using MSNPs/GO as signaling labels. Reprinted from [82] with permission.

The use of label-free detection minimizes complicated and time-consuming procedures, and also facilitates the design of miniaturized highly portable equipment for POC devices. For instance, the detection of the important breast cancer biomarker CA 15-3 was accomplished by Li et al. [81] by preparing a sensitive and reliable immunosensor involving the use of GCEs modified with N-doped graphene sheets (NGS) as a platform for electrochemical detection. Anti-CA 15-3 antibodies were covalently attached to NGS surface through the carboxylic groups of this material and [Fe(CN)_6_]^3−/4−^ was used as the redox probe. In this configuration, the high conductivity of the modified electrode in which NGS significantly promoted electron transfer resulted in a greatly amplified signal with no need for tracer labels. Another immunosensor for the detection of CA 15-3 was fabricated by using click chemistry [82]. As is known, copper(I) catalyzed azide-alkyne cycloaddition (“click” chemistry) has emerged as an efficient strategy for biomolecule immobilization. Some advantages for this reaction are the high yields, no by-products, and moderate conditions, as well as the possibility to be performed in aqueous and physiological conditions. In this work, a copper-catalyzed 1,3-dipolar cycloaddition reaction was used to prepare magnetic silica nanoparticles/GO composites (MSNPs/GO) by conjugation of azide-functionalized magnetic silica nanoparticles to alkynyl-functionalized GO (Figure 9). The resulting nanomaterial exhibited a high peroxidase-like activity and, compared with conventional enzyme labels, it exhibited the advantages of environmental friendliness and improved stability. For construction of a label-free immunosensor, disposable screen-printed electrodes modified with GO were used to covalently link monoclonal anti-CA 15-3 antibody, with MSNPs/GO enzyme-like composites acting as the signal labels.

An electrochemical immunosensor for quantification of p53 was developed using streptavidin-functionalized AuNPs (Strept-AuNPs) and thiolated graphene oxide (SH-GO). Biotinylated capture anti-p53 antibodies were immobilized on the electrode surface via Strept-AuNPs and a sandwich immunoassay was performed using secondary anti-p53 labeled with HRP. The electrocatalytic reduction of THI in the presence of H_2_O_2_ was amplified by linked HRP under optimized conditions, this providing a high sensitivity. Moreover, the use of GO and Strept-AuNPs not only improved the electron transfer rate, but also increased the amount of anti-p53 capture antibody. The proposed immunosensor was validated by detection of p53 protein in spiked human serum samples and also in normal and cancerous human skin fibroblast cells [83].

Non-invasive strategies for sample collection are recommended tools for detection of human diseases [84]. Thus, various biological fluids, such as saliva, urine, sweat and tears, have been proposed as valuable samples for specific analysis. Among them, saliva has some advantages mainly derived from its easy sampling and the relatively abundant diagnostic biomarkers [85]. For instance, the use of this sample is of high interest in detecting oral cancer. CYFRA-21-1 (Cytokeratin-19, CA-19), the smallest human cytokeratin protein, is a highly sensitive biomarker for non-small cell lung cancer (NSCLC) [50] and is also present at high concentrations in saliva of oral cancer patients [86]. With the objective of specifically detecting this marker, an immunosensor was prepared immobilizing anti-CYFRA-21-1 onto an ITO substrate coated by rGO decorated with zirconia nanoparticles (ZrO_2_NPs) and APTES (3-aminopropyl-triethoxy-silane) [39]. Improved heterogeneous electron transfer was observed for the resulting APTES/ZrO_2_NPs/rGO/ITO electrode with respect to that prepared in the absence of rGO and, moreover, APTES amino groups proved adequate in binding the capture antibody. All this contributed to making it possible to determine the clinical concentrations of CYFRA-21-1 in saliva by means of a label free scheme using DPV in the presence of [Fe(CN)_6_]^3−/4−^.

**Figure 10:**
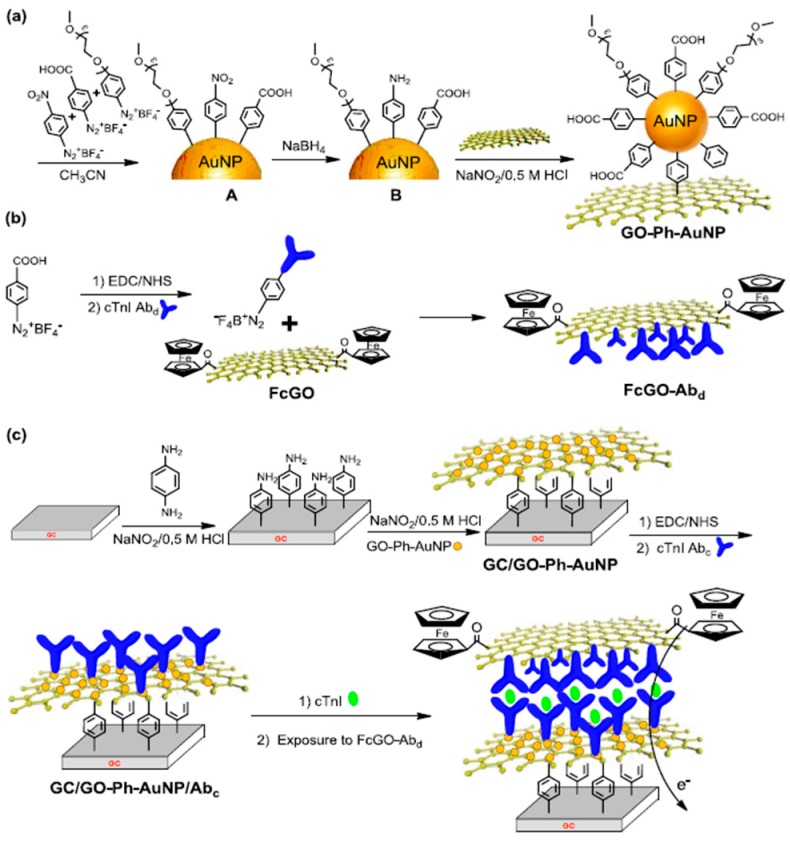
Scheme of the preparation of GO-Phe-AuNP nanocomposites (AuNP A indicates AuNP modified with mixed layers of PEG, 4-carboxyphentl and 4-nitrophenyl; AuNP B indicates AuNP modified with mixed layers of PEG, 4-carboxyphentl and 4-aminephenyl) (**a**); the preparation of ferrocene-GO-modified detection antibody (**b**); and the fabrication of the cTnI immunosensor (**c**). Reprinted from [87] with permission.

For the detection of cTnI cardiovascular biomarker in the early diagnosis of myocardial infarction, a stable label-free amperometric immunosensor was recently reported involving AuNPs and GO nanocomposites covalently anchored onto GCEs by aryldiazonium salt coupling using 4-phenylenediamine (Figure 10). AuNPs were previously modified with mixed layers of 4-nitrophenyl, 4-carboxyphenyl, and polyethylene glycol (PEG). These functions were used for linking AuNPs to GO surface and to covalently immobilize the capture antibody. Moreover, the presence of PEG groups helped to avoid nonspecific adsorptions. The resulting platform (GO-Phe-AuNPs) had a large surface and provided a high stability to the sensing interface. Furthermore, a signal amplification strategy for the determination of cTnI was implemented by immobilizing the detector antibody on GO tailored with ferrocene, thus functioning as the signal reporter [87].

Various impedimetric immunosensors for cTnI were also developed [60,88,89] (Table 3). Signal et al. [89] used functionalized PtNPs-decorated GR deposited onto GCE. A GR monolayer was firstly transferred to the electrode followed by electrochemical treatment to obtain the so-called electroactive graphene (EG) by creation of defects and oxidized groups that enhance the electrochemical behavior. Next, surface modification of EG/GCE was made with MPA capped-PtNPs using 1-pyrenemethylamine (PMA) interlinker. Anti-cTnI antibodies were covalently attached to MPA-PtNPs through the carboxyl group, and the resulting anti-cTnI-MPA-PtNPs-PMA-EG/GCE immunosensor was utilized to detect different concentrations of antigen using EIS by measurement of changes in the R_CT_ in the presence of [Fe(CN)_6_]^3−/4−^.

## 5. General Conclusions and Future Prospects

Electrochemical affinity biosensors using nanomaterials may offer various advantages for enhancing and superseding the capabilities of current analytical methodologies by permitting rapid and highly accurate analysis. Besides the most common carbon nanomaterials used for the construction of electrochemical sensors (CNTs and graphene), in recent years, new carbon nanoforms have appeared as suitable means for nanostructuration of electrode surfaces and biomolecules’ immobilization. Examples are graphene quantum dots, fullerenes, carbon nanohorns, double-walled carbon nanotubes and carbon nanoparticles. These nanomaterials, though, have scarcely been employed in the design of electrochemical biosensors for biomarkers’ detection, but just a few examples have demonstrated some specific properties of great interest [90,91].

Despite much progress, this field is still new, and there are many points yet to be addressed in the development of electrochemical biosensors implying the use of carbon nanomaterials as modifiers of the sensing platforms in order to further improve their analytical performance. All applications discussed are in vitro or lab on chip applications. Apart from being compatible with the target biomolecule and specific physiological environments required by many complex biological systems, these nanomaterials-based biosensors need to possess a certain degree of biocompatibility in order to develop next-generation biosensors also suitable for in vivo studies. Moreover, the possibility of tuning the binding sites of these nanomaterials and their properties for the simultaneous determination of different analytes is highly desirable. Furthermore, apart from exploring widely different nanomaterial combinations, it is important to solve the major issue of nanomaterial aggregation. Beyond these requirements, and taking into account the growing demand for high throughout and multiplexed assays, other challenges include the development of electrochemical instrumentation able to perform multiplexed and parallel measurements while retaining their low cost and portability and still being able to perform remote wireless detection in real clinical or field environments.

## Figures and Tables

**Table 1:** Electrochemical DNA biosensors for diagnosis using carbon nanomaterials as electrode modifiers.

Electrode	Type of Carbon Nanomaterial	Analyte	Sample	Technique	L.R.	LOD	Reference
Au electrode	PAMAM G4-MWCNTs	human cellular prions protein (PrP^C^)	spiked blood plasma	CV	1 pM–10 μM	0.5 pM	[26]
Au electrode	MWCNTs-PPy-PAMAM G4	*rpoB* gene of *Mycobacterium tuberculosis*	real PCR samples	SWV	1 fM–10 pM (15 nts synthetic target), 1–100 fM (75 nts synthetic targets)	0.3 fM (for both target DNAs)	[8]
Au electrode	MWCNTs	Hepatitis C viral DNA and *Mycobacterium tuberculosis* genomic DNA	*Mycobacterium tuberculosis* (H37Rv) rpoB allele in DNA extracted from clinical isolates	EIS	0.1 fM–1 pM (synthetic oligonucleotide from Hepatitis C virus)	7 fM	[16]
GCE	GO	cancer-related *BRCA1* gene	-	chronoamperometry	1 fM–1 nM	1 fM	[18]
GCE	graphene/AuNR/PT	HPV DNA	serum samples	DPV	1.0 × 10^−13^–1.0 × 10^−10^ M	4.03 × 10^−14^ M	[10]
CILE	ZrO_2_/graphene	*S. aureus* thermonuclease *nuc* gene	PCR samples	DPV	1.0 × 10^−^^13^–1.0 × 10^−^^6^ mol·L^−^^1^	3.23 × 10^−^^14^ M	[15]
GCE	SWCNTs/CFGO	*Vibrio parahaemolyticus thermolabile hemolysin* (*tlh*) gene	-	DPV	1 × 10^−6^–1 × 10^−13^ mol·L^−1^	7.27 × 10^−14^ M	[11]
GCE	graphene-MSGNs	DNA sequences correlated to Alzheimer, thrombin, ATP	-	DPV	-	-	[21]
GCE	GSHs	mutated *apolipoprotein E* gene associated with Alzheimer’s disease	-	DPV	-	-	[12]

CILE: carbon-ionic liquid electrode; CV: cyclic voltammetry; DPV: differential pulse voltammetry; EIS: electrochemical impedance spectroscopy; GCE: glassy carbon electrode; GSHs, graphene@mesoporous silica hybrids; GO: graphene oxide; GR/AuNR/PT: GR/Au nanorod/polythionine; HPV: human papillomavirus; L.R.: linear range; MSGNs: mesoporous materials; PAMAM, polyamidoamine; PPy: polypyrrole; PrP^C^: cellular prions protein; SWCNTs/CFGO: SWCNTs-carboxyl functionalized GO; SWV: square-wave voltammetry; ZrO_2_/GR: zirconia-graphene.

**Table 2:** Electrochemical immunosensors involving CNTs-modified platforms for the determination of cancer and cardiovascular biomarkers.

Electrode	Analyte	Immunoassay	Technique	Linear Range	LOD	Sample	Reference
Cancer biomarkers							
AuNPs-HDT-AuNPs/MW-CILE	HER2	Direct with immobilized anti-HER	EIS	10–110 ng·mL^−1^	7.4 ng·mL^−1^	serum	[43]
MWCNTs-ZnONF/GCE	CA-125	Direct with immobilized anti-CA-125. [Fe(CN)_6_]^3−/4−^ as the redox probe	DPV	0.001–1000 U·mL^−1^	0.00113 U·mL^−1^	spiked serum	[45]
THI/MWCNTs/IL/GCE	PSA	Sandwich with immobilized anti-PSA and HRP-Ab2	DPV	0.2–1.0 ng·mL^−1^ 1–40 ng·mL^−1^	20 pg·mL^−1^	prostate tissue, serum	[47]
MWCNTs/IL/Chit/GCE	PSA	Direct (EIS) with immobilized anti-PSA-AuNPs-PAMAM, or sandwich (DPV) with HRP-Ab2	EIS DPV	up to 25 ng·mL^−1^ up to 80 ng·mL^−1^	0.5 pg·mL^−1^ 1 pg·mL^−1^	serum	[48]
MWCNTs/DAH/GCE	PSA	Sandwich with immobilized anti-PSA and Ab2-AuNPs-FcH	DPV	0.01–100 ng·mL^−1^	5.4 pg·mL^−1^	spiked human serum	[49]
SWCNTs/GCE	NSE	Indirect competitive with immobilized NSE, anti-NSE, and AP-anti-IgG/AuNPs	DPV	0.1–2000 ng·mL^−1^	0.033 ng·mL^−1^	clinical serum specimens	[51]
SWCNTs/Nafion/Fe(OH)_x_/PG	IL-6	Sandwich with immobilized anti-IL6 and multi-HRP-MWCNTs-labeled-Ab2	amperom	0.5–30 pg·mL^−1^	0.5 pg·mL^−1^	calf serum	[52]
SWCNTs/TMCS-MPS/graphene/GCE	AFP	Direct with immobilized anti-AFP. FCA as the redox probe	DPV	0.1–100 ng·mL^−1^	0.06 ng·mL^−1^	serum	[53]
Au/PDDA/MWCNTs/(PSS/PDDA/MWCNTs)_2_/GCE	CEA	Direct with immobilized anti-CEA. [Fe(CN)_6_]^3−/4−^ as the redox probe	CV	0.1–2.0; 2.0–160 ng·mL^−1^	0.06 ng·mL^−1^	serum	[55]
pTHI/PA6/MWCNTs/GCE	p53	Sandwich with immobilized anti-p53 and HRP-Ab2	DPV	0.002–2 ng·mL^−1^	<1 pg·mL^−1^	-	[57]
Cardiovascular biomarkers							
PtNPs(MPA)/G-MWCNTs	cTn1	Direct with immobilized anti-cTn1	EIS	0.001–10 ng·mL^−1^	1.0 pg·mL^−1^	human serum	[61]
SU-8/MWCNTs/GCE	Mb, cTn1, CK-MB	Direct with immobilized anti-Mb, or anti-cTnI, or anti-CK-MB	EIS	1–50 (Mb); 0.1–10 (cTnI); 10–10,000 (CK-MB) ng·mL^−1^	0.1 (Mb; cTnI); 1 ng·mL^−1^ (CK-MB)	-	[67]
CNTs/PEI/AuE	cTnT	Sandwich with immobilized anti-cTnT and Ab2-HRP	amperom.	0.1–10 ng·mL^−1^	0.033 ng·mL^−1^	human serum	[62]
NH_2_-MWCNTs/SPE	cTnT	Direct with immobilized anti-cTnT. [Fe(CN)_6_]^3−/4−^ as the redox probe	DPV	0.0025–0.5 ng·mL^−1^	0.0035 ng·mL^−1^	serum	[63]
MWCNTs/SPE	Mb	Direct with immobilized anti-Mb	EIS	0.1–90 ng·mL^−1^	0.08 ng·mL^−1^	serum	[70]
MWCNTs/THI/AuNPs/GCE	MPO	Direct with immobilized anti-MPO. THI as the redox probe.	CV	2.5–125 ng·mL^−1^	1.425 ng·mL^−1^	human serum	[65]
p*o*PD-MWCNTs-IL/ITO	MPO	Direct with immobilized anti-MPO	amperom.	0.2–23.4 ng·mL^−1^ 23.4–300 ng·mL^−1^	0.05 ng·mL^−1^	human serum	[66]
Cyst/SPAuE	oxLDL	Direct with immobilized anti-oxLDL	SWV EIS	3–10.5 μg·mL^−1^ 0.5–18.0 μg·mL^−1^	0.22 μg·mL^−1^	serum	[68]
p-MWCNTs@Chit/Nafion/THI/Au NPs/GCE	netrin 1	Direct with immobilized anti-netrin 1	DPV	0.09–1800 pg·mL^−1^	30 fg·mL^−1^	serum	[72]
AuNPs-CNTs/AuE	NT-proBNP	Sandwich with immobilized anti-NT-proBNP and HRP-Ab2-AuNCs	CV	0.02–100 ng·mL^−1^	6 pg·mL^−1^	-	[74]

FCA, ferrocene carboxylic acid; MW-CILE, multi-walled carbon nanotubes-ionic liquid paste electrode; DAH, 1,7-diaminoheptane; FcH, 6-ferrocenylhexanethiol; HDT, 1,6-hexanedithiol; Mb, myoglobin; MPA, 3-mercaptopropionic; MPO, myeloperoxidase; MPS, mesoporous silica; PA6, a type of Nylon; PDDA, polydiallyldimethyl ammonium; PG, pyrolytic graphite; p*o*PD, poly(*o*-phenylenediamine); PSS, poly(sodium-p-styrene-sulfonate); THI, thionine; IL, ionic liquid; TMCS, trimethylchlorosilane; ZnONF, ZnO nanofibers; ITO, indium tin oxide.

**Table 3:** Electrochemical immunosensors involving graphene-modified platforms for the determination of cancer and cardiovascular biomarkers.

Electrode	Analyte	Immunoassay	Technique	Linear Range	LOD	Sample	Reference
Cancer biomarkers							
Strept-AuNPs-SH-GO/GCE	p53	Sandwich with immobilized Biotin-anti-p53 and HRP-Ab2	DPV	0.2–2 pmol·L^−1^ 2–200 pmol·L^−1^	30 pmol L^−1^	human serum	[83]
SnO_2_/AuNPs/rGO/GCE	AFP	Direct with immobilized anti-AFP. [Ru(NH_3_)_6_]^3+^ as the redox probe.	DPV	0.02–50 ng·mL^−1^	0.01 ng·mL^−1^	spiked serum	[79]
AuNPs/NB/rGO/GCE	CEA	Direct with immobilized anti-CEA. Nile Blue (NB) as the redox probe	DPV	0.001–40 ng·mL^−1^	0.00045 ng·mL^−1^	spiked serum	[80]
GO-SPCE	CA 153	Sandwich with immobilized anti-CA153 and Ab2-ZnFe_2_O_4_/GO	DPV	10^−3^–200 U·mL^−1^	2.8 × 10^−4^ U·mL^−1^	spiked serum	[82]
3D-rGO/AuNPs/	PSA	Direct with immobilized anti-PSA. [Fe(CN)_6_]^3−/4−^ as the redox probe	CV	up to 10 ng·mL^−1^	0.59 ng·mL^−1^	-	[76]
APTES/ZrO_2_-rGO/ITO	CYFRA-21-1	Direct with immobilized anti-CYFRA-21-1. [Fe(CN)_6_]^3−/4−^ as the redox probe	DPV	2–22 ng·mL^−1^	0.122 ng·mL^−1^	saliva	[39]
Ag/Au–DN–graphene/GCE	CEA	Sandwich with immobilized anti-CEA and Ab2-Ag/Au–DN-graphene	Amperometry	0.01–1200 ng·mL^−1^	8 pg·mL^−1^	spiked human plasma	[75]
AuPdPtNPs/rGO/TEPA/GCE	NMP22	Direct with immobilized anti-NMP22. [Fe(CN)_6_]^3−/4−^ as the redox probe	DPV	0.040–20 U·mL^−1^	0.01 U·mL^−1^	urine	[78]
Cardiovascular biomarkers							
PrGO/GCE	cTnI	Direct with immobilized anti-TnI	EIS	0.1–10 ng·mL^−1^	0.07 ng·mL^−1^	-	[88]
PtNPs(MPA)/EG/GCE	cTnI	Direct with immobilized anti-TnI	EIS	0.01–10 ng·mL^−1^	4.2 pg·mL^−1^	-	[89]
AuNPs-Ph-GO/GCE	cTnI	Direct with immobilized anti-cTnI-Fc-GO. [Fe(CN)_6_]^3−/4−^ as the redox probe	SWV	0.05–3 ng·mL^−1^	0.05 ng·mL^−1^	spiked serum	[87]
PtMPA/GR-MWCNTs/GCE	cTnI	Direct with immobilized anti-TnI	EIS	0.001–10 ng·mL^−1^	0.001 ng·mL^−1^	-	[61]

DN, 1,5-diaminonaphthalene; EG, electroactive graphene; MPA, 3-mercaptopropionic acid; PMA, 1-pyrenemethylamine; PrGO, porous graphene oxide; TEPA, tetraethylene pentamine.
